# Effect of Calcination Temperatures on Crystallite Size, Particle Size, and Antimicrobial Activity of Synthesized MgO and Its Cytotoxicity

**DOI:** 10.3390/ijms26104868

**Published:** 2025-05-19

**Authors:** Gopinath Kasi, Nattan Stalin, Pornchai Rachtanapun, Kittisak Jantanasakulwong, Joshua Nizel Halder, Suphat Phongthai, Patnarin Worajittiphon, Jongchul Seo, Sarinthip Thanakkasaranee

**Affiliations:** 1 Faculty of Agro-Industry, Chiang Mai University, Mae-Hea, Mueang, Chiang Mai 50100, Thailand; gopiscientist@gmail.com (G.K.); pornchai.r@cmu.ac.th (P.R.); kittisak.jan@cmu.ac.th (K.J.); suphat.phongthai@cmu.ac.th (S.P.); 2 Department of Life Science, Gachon University, Seongnam 13120, Gyeonggi-do, Republic of Korea; nattanstalin@gmail.com; 3 Center of Excellence in Agro Bio-Circular-Green Industry, Faculty of Agro-Industry, Chiang Mai University, Chiang Mai 50100, Thailand; 4 Center of Excellence in Materials Science and Technology, Chiang Mai University, Chiang Mai 50200, Thailand; patnarin.w@cmu.ac.th; 5 Office of Research Administration, Chiang Mai University, Chiang Mai 50200, Thailand; joshua.halder@cmu.ac.th; 6 Faculty of Agriculture, Chiang Mai University, Chiang Mai 50200, Thailand; 7 Department of Chemistry, Faculty of Science, Chiang Mai University, Chiang Mai 50200, Thailand; 8 Department of Packaging & Logistics, Yonsei University, 1 Yonseidae-gil, Wonju-si 26493, Gangwon-do, Republic of Korea; jcseo@yonsei.ac.kr

**Keywords:** MgO nanoflakes, calcination temperature, particle size, antimicrobial activity, cytotoxicity

## Abstract

The development of antimicrobial agents with excellent antimicrobial activity, high thermal stability, and non-toxic properties is an essential demand in food packaging, pharmaceutical, and biomedical applications. In the present study, MgO nanoflakes were synthesized via the co-precipitation method by varying calcination temperatures at 400, 500, and 600 °C. The effect of calcination temperature on the morphology, thermal stability, antimicrobial activity, and cytotoxicity of the MgO nanoflakes was analyzed. All synthesized MgO samples showed a Face-Centered Cubic (FCC) structure. As the calcination temperature increased, the MgO crystallite and particle sizes increased, whereas the surface area decreased. In addition, the MgO synthesized from higher calcination temperatures showed higher thermal stability and crystallinity. The MgO synthesized from the lower calcination temperatures (400 and 500 °C) showed superior antimicrobial activity against *Escherichia coli* (100% R) and *Staphylococcus aureus* (100% R) than that from the higher calcination temperature (600 °C). The cytotoxicity test demonstrates slight cytotoxicity in MgO-400 °C at concentrations ranging from 100 to 200 µg/mL. Interestingly, MgO-500 °C and MgO-600 °C demonstrate biocompatibility and exhibit non-cytotoxic effects, respectively, highlighting their potential for practical applications in active food packaging and biomedical fields.

## 1. Introduction

Microbial contamination continues to be a major constraint in the global food supply chain, particularly fresh produce, contributing to significant public health risks, economic losses, and the deterioration of food quality. Fruits and vegetables have been recognized by the World Health Organization (WHO) and the Food and Agriculture Organization (FAO) as principal vehicles in the transmission of foodborne illnesses associated with Shiga-toxin-producing *Escherichia coli* (STEC) [1]. In addition, the WHO reported that contaminated food leads to 600 million illnesses and 420,000 deaths globally each year, with children under five accounting for 30% of the fatalities [2,3,4]. Traditional preservation strategies, such as refrigeration and thermal treatment, offer limited efficacy in providing sustained antimicrobial protection during post-processing handling, storage, and distribution [1,5]. The development of alternative antimicrobial agents is in high demand for active antimicrobial packaging systems to address such issues and ensure food safety, which is related to the United Nations Sustainable Development Goals (SDGs), especially SDG3, which deals with good health and well-being.

Antimicrobial agents such as essential oils and plant extracts have been used in active food packaging due to their natural and non-toxic properties [6]. However, the drawbacks of such organic-based antimicrobial agents include rapid volatility, fast degradation over time, thermal instability [7], and susceptibility to degradation during fabrication of antimicrobial packaging via thermally conventional processes such as blown film extrusion and cast film extrusion, limiting the potential for practical applications. These highlight the demand for alternative antimicrobial agents with safe and high thermal stability. Metal and metal oxide nanoparticles have emerged as promising antimicrobial agents in active food packaging due to their superior antimicrobial properties, thermal stability, and extended efficacy compared to organic antimicrobial agents [8]. Silver (Ag), zinc oxide (ZnO) [9], and titanium dioxide (TiO_2_) nanoparticles [10] have been widely studied for their potential applications in food packaging. Despite their broad-spectrum antimicrobial activity, the cytotoxicity associated with Ag, especially at higher concentrations, limits their safe application [11]. Additionally, the required photocatalytic activity of metal oxides like ZnO and TiO_2_ for their antimicrobial activity raises concerns regarding their stability and performance under varying environmental conditions [9,10] in active food packaging applications.

Among these metal and metal oxide nanoparticles, magnesium oxide (MgO) nanoparticles have gained significant attention owing to their stable physicochemical properties, unique biocompatibility, and strong antibacterial activity, with no photocatalytic activity required in their antimicrobial mechanism [12]. In addition, MgO nanoparticles exhibit exceptional stability, high resistance to extreme temperatures, and elevated ionization potential, making them particularly well-suited for applications in food packaging [13], wound dressings [14], and biomedical technologies [15]. The effectiveness of MgO nanoparticles has been determined by their synthesis method, which influences their structural, morphological, and functional characteristics. Several synthesis techniques, including co-precipitation [16], hydrothermal [17], sol–gel [17], and green synthesis [18,19], have been explored to control nanoparticle size, morphology, and stability. The co-precipitation method, in particular, offers notable advantages such as simplicity, cost-effectiveness, and scalability, enabling the production of nanoparticles with a uniform composition under ambient conditions [20]. Thus, the research study for effective and safe antimicrobial alternatives has intensified interest in MgO nanoparticles as a promising candidate. MgO nanoparticles function by disrupting bacterial membranes and generating reactive oxygen species (ROS), key mechanisms that contribute to their antimicrobial efficacy. However, despite their promising potential, several challenges persist in comprehensively understanding their antibacterial properties, cytotoxicity, and overall biocompatibility [12,21]. Comparative studies evaluating the antimicrobial effectiveness of MgO nanoparticles with different sizes, synthesized using the co-precipitation method at varying calcination temperatures, against Gram-positive and Gram-negative bacterial strains, as well as their impact on the RAW 264.7 macrophage cell line, are limited. In addition, in the literature, the antimicrobial activity of MgO nanoparticles has been mostly studied via the zone of inhibition (ZOI), which has several drawbacks. The ZOI is unsuitable for non-diffusing or surface-bound antimicrobials and provides limited quantification compared to the other methods (e.g., broth dilution). Moreover, the ZOI is influenced by agar composition and diffusion, leading to variability. In addition, the ZOI may yield false negatives for effective but non-leaching materials.

Addressing these knowledge gaps is essential to ensure the safe and effective utilization of MgO in practical applications. This study aims to synthesize MgO nanoflakes using the co-precipitation method with a controlled stirring time of 90 min at different calcination temperatures (400 °C, 500 °C, and 600 °C). The effect of calcination temperatures on particle size, crystallite size, surface area, and thermal stability of synthesized MgO nanoflakes was characterized. In addition, the effect of calcination temperatures on the antibacterial activity of MgO nanoflakes with different particle sizes and surface areas was evaluated using the broth dilution method, providing accurate results and enabling direct comparison of antimicrobial potency. Furthermore, the cytotoxicity of MgO nanoflakes with different particle sizes and surface areas was investigated using the RAW 264.7 cell line, ensuring an alternative, safe antimicrobial agent. The findings from this study are expected to advance the development of MgO nanoflakes for practical applications in food packaging (e.g., antimicrobial coating, antimicrobial films, etc.) and biomedical fields (e.g., wound dressing, tissue scaffolds, etc.), ultimately contributing to enhanced public health and safety.

## 2. Results and Discussion

### 2.1. Crystallographic Structure and Crystallite Size

**Figure 1:**
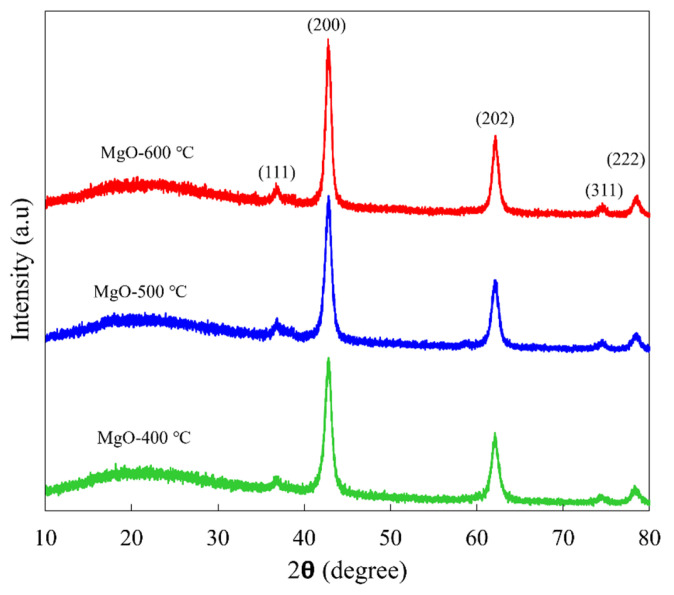
XRD patterns of synthesized MgO at different calcination temperatures of 400, 500, and 600 °C.

The effect of calcination temperatures (400, 500, and 600 °C) on the crystallographic structure and crystallite size of synthesized MgO nanoflakes was investigated using XRD. As shown in Figure 1, all synthesized MgO samples showed diffraction peaks at 2*θ* values of 36.9, 42.9, 62.2, 74.4, and 78.4° corresponding to the (1 1 1), (2 0 0), (2 2 0), (3 1 1), and (2 2 2) planes of a Face-Centered Cubic (FCC) structure, according to JCPDS file no. 89-7746. No additional peaks were observed in the XRD pattern, confirming the high purity of all synthesized MgO nanoflakes. Interestingly, the peak intensity of MgO slightly increased as the calcination temperature increased from 400 to 500 and 600 °C. A higher peak intensity in the XRD pattern indicates a larger crystallite size. As shown in Table 1, the average crystallite size was 8.80, 8.88, and 10.97 nm for the synthesized MgO-400 °C, MgO-500 °C, and MgO-600 °C, respectively, which was estimated using Scherrer’s formula. This trend is consistent with previous studies. Diana et al. [22] reported that MgO nanoparticles annealed at 500, 600, and 700 °C for 3 h exhibited a progressive increase in crystallite size, as confirmed by XRD analysis, with values of 22, 25, and 29 nm, respectively. Similarly, Hegde et al. [23] observed that the crystallite size of MgO nanoparticles increased from 8.26 nm at 550 °C to 18.95 nm at 750 °C due to grain agglomeration, emphasizing the role of thermal processing in enhancing structural order and crystallinity. Sundrarajan et al. [24] also reported that the crystallite size increased from 4.6 nm at 300 °C to 8.5 nm at 500 °C and 13.3 nm at 700 °C, attributing nanoparticle aggregation driven by inter-particle electrostatic forces. The current work demonstrates that at a lower temperature of 400 °C, the incomplete precursor decomposition leads to structural defects, disorder, and strain. Increasing the temperature to 500 °C and 600 °C enhances atomic arrangement, improving crystallinity. The higher temperatures facilitate atomic diffusion, reducing lattice defects and promoting a more ordered structure. These findings collectively highlight the significant influence of calcination temperature on the crystallite size and structural evolution of MgO nanoflakes.

### 2.2. Morphology and Element Composition

**Figure 2:**
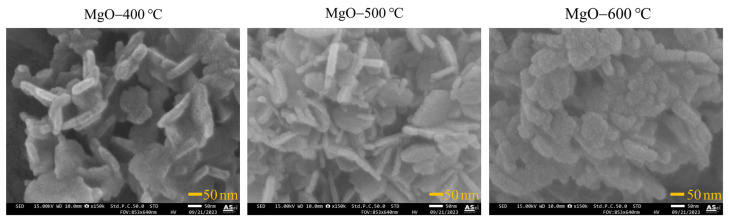
SEM images of synthesized MgO at different calcination temperatures of 400, 500, and 600 °C.

**Figure 3:**
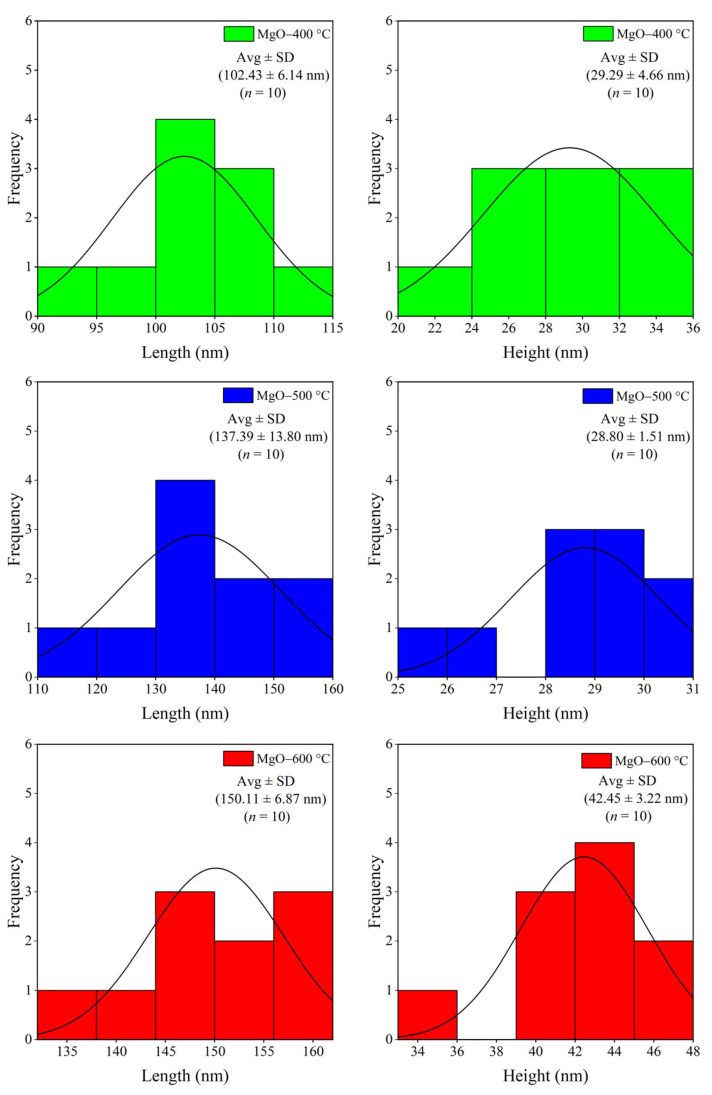
The average particle size of synthesized MgO at different calcination temperatures of 400, 500, and 600 °C was determined using SEM analysis.

**Figure 4:**
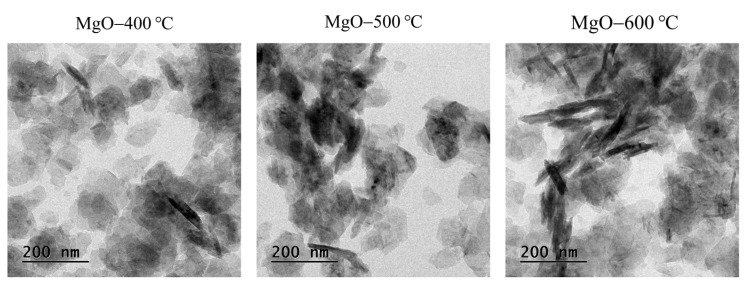
TEM images of synthesized MgO at different calcination temperatures of 400, 500, and 600 °C.

**Figure 5:**
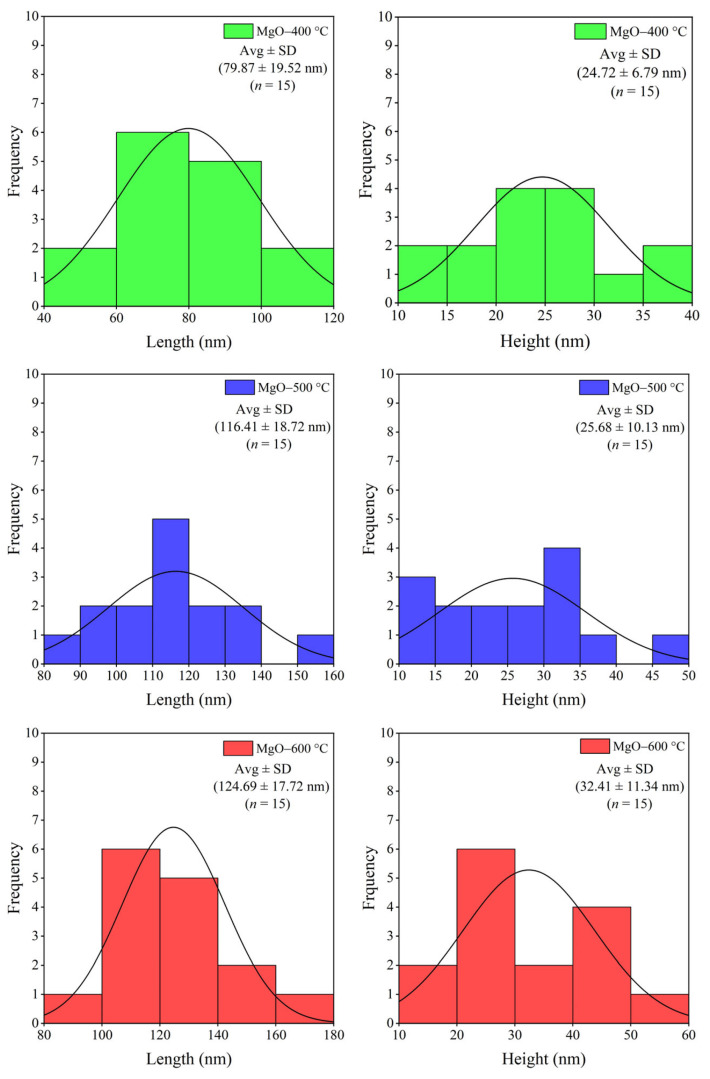
The average particle size of synthesized MgO at different calcination temperatures of 400, 500, and 600 °C was determined using TEM analysis.

The effect of calcination temperatures (400, 500, and 600 °C) on the shape and particle size of the synthesized MgO was investigated using SEM and TEM. As shown in SEM images (Figure 2), all synthesized MgO samples showed a nanoflake structure. At the low calcination temperature, the shape (corners and edges) of MgO-400 °C was not sharp, which is attributed to a lower crystalline phase. At the optimal calcination temperature, the shape of MgO-500 °C was sharper due to its well-formed crystalline structure. At higher calcination temperatures (600 °C), the sharpness of the MgO nanoflakes diminished, likely due to the growth of MgO grains and the aggregation of MgO particles. The particle size of the synthesized MgO is presented in Figure 3. As the calcination temperature increased, the particle size of the synthesized MgO nanoflakes also increased from 102 nm × 29 nm to 137 nm × 28 nm and 150 nm × 42 nm (length × height) for MgO-400 °C, MgO-500 °C, and MgO-600 °C, respectively. The morphology of all synthesized MgO nanoflakes was also verified by TEM analysis, as shown in Figure 4, confirming the nanoflake structure of all synthesized MgO samples. The particle size of synthesized MgO increased with the increase in calcination temperatures, which is consistent with the SEM results. The particle sizes of MgO-400 °C, MgO-500 °C, and MgO-600 °C were 80 nm × 25 nm, 116 nm × 26 nm, and 125 nm × 32 nm, respectively, as shown in Figure 5. The average particle size of MgO investigated by SEM was larger than that studied using TEM. The SEM images showed a larger size of MgO nanoflakes owing to the additional layer of gold grains on the surface of MgO particles, affected by gold sputtering for specimen preparation before analysis. The increase in particle size of the synthesized MgO nanoflakes correlates with the trend observed in the crystallite size, as shown in Table 1. At elevated calcination temperatures, the nanograins tend to merge into larger particles due to the high surface energy. In addition, a crystallite is defined as a single crystal with a long-range atomic arrangement, whereas a particle generally comprises several crystallites. Therefore, the increase in MgO particle size with higher calcination temperatures is also attributed to the larger crystallite size. This observation is in agreement with the XRD and BET results as well as the previous reports. Huang et al. [25] showed that the particle size of MgO derived from amorphous magnesite increased with the rise in calcination temperatures. The element composition of the synthesized MgO nanoflakes is shown in Table 2. The SEM-EDS analysis demonstrates no significant change in the element composition (Mg/O) of the synthesized MgO with the rise in calcination temperatures.

**Table 1:** Average crystal size and BET surface area of synthesized MgO at different calcination temperatures of 400, 500, and 600 °C.

Sample	Average Crystal Size (nm)	BET Surface Area (m^2^/g)
MgO-400 °C	8.80	127.88
MgO-500 °C	8.88	88.06
MgO-600 °C	10.97	86.45

### 2.3. Surface Area of MgO

The influence of calcination temperatures on the specific surface area of synthesized MgO nanoflakes is depicted in Table 1. As the calcination temperature increased from 400 to 600 °C (calcination for 3 h), the specific surface area of the synthesized MgO decreased from 127.88 to 88.06 and 86.45 m^2^/g for MgO-400 °C, MgO-500 °C, and MgO-600 °C, respectively. At elevated calcination temperatures, nanoflakes are prone to coalescing or agglomerating through the sintering process. This phenomenon is driven by the increased thermal energy, which facilitates the diffusion of atoms on the MgO particle surfaces, promoting the fusion of individual particles. Consequently, smaller MgO nanoflakes combine to form larger structures, leading to a decrease in the specific surface area. In addition, the high calcination temperatures can induce the growth of crystallites within MgO nanoflakes, as shown in Table 1. As these crystallites enlarge, the surface area is reduced due to the diminishing presence of high-energy sites, such as edges and corners, in favor of more thermodynamically stable, low-energy crystal planes [26,27]. The BET surface area result of the synthesized MgO nanoflakes is consistent with the XRD and SEM results. However, Huang et al. [25] found that the surface area of the MgO calcined from the amorphous magnesite depends on the calcination temperatures. The specific surface area of MgO increased as the calcination temperature rose from 400 to 600 °C, but it decreased when the temperature was increased from 700 to 1150 °C after 30 min of calcination. They noted that between 400 °C and 600 °C, the specific surface area of MgO increased with temperature, as the decomposition of amorphous magnesite generates a porous structure. After the complete decomposition of magnesite at 600 °C, the specific surface area of pure MgO decreased with the rise in temperature, likely due to the aggregation and coalescence of MgO nanocrystals.

### 2.4. Thermal Stability

**Figure 6:**
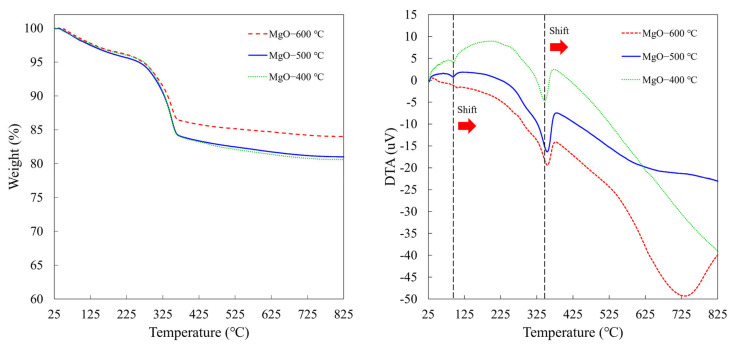
TGA and DTA curves of synthesized MgO at different calcination temperatures of 400, 500, and 600 °C.

In general, Mg(OH)_2_ can be converted to MgO via a calcination process up to 400 °C for 3 h. In this study, the effect of calcination temperatures on the thermal stability of the synthesized MgO nanoflakes was investigated using TGA. Figure 6 shows the TGA and DTA of the synthesized MgO-400 °C, MgO-500 °C, and MgO-600 °C. All synthesized MgO nanoflakes displayed a two-step decomposition process, occurring between 50 and 150 °C and 200 and 800 °C. The first weight loss corresponded to the release of water absorbed on the MgO surface, while the second weight loss was due to the evaporation of CO_2_ adsorbed on the MgO surface [28]. Among synthesized MgO nanoflakes, MgO-600 °C showed the lowest weight loss. Furthermore, the decomposition temperature of MgO increased with the calcination temperature, as evidenced by the rightward shift of the endothermic peak in the DTA curves, from 347 to 355 °C. This suggests that MgO calcined at 600 °C exhibits the highest thermal stability. At higher calcination temperatures, MgO undergoes crystallization, transitioning from an amorphous or poorly ordered structure to a more ordered, crystalline form (higher crystallinity) [29]. This crystalline structure is more thermodynamically stable and less prone to decomposition or phase transitions at elevated temperatures. Furthermore, the larger crystallite and particle sizes enhance the thermal stability of MgO nanoflakes because their lower surface-to-volume ratio reduces their susceptibility to further structural changes at elevated temperatures. This TGA result aligns with the observations from XRD, SEM, and BET analyses.

### 2.5. Antimicrobial Activity

**Figure 7:**
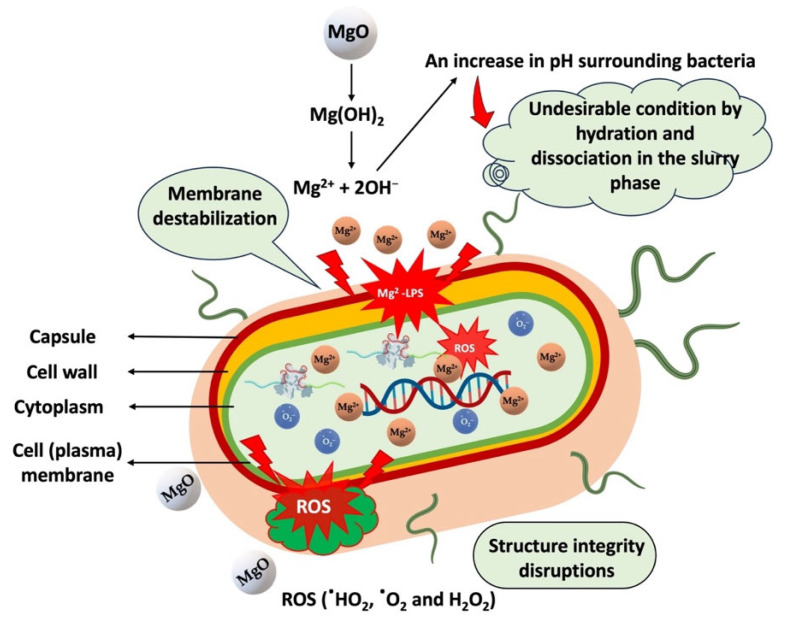
Antimicrobial mechanism of MgO nanoflakes.

Active antimicrobial food packaging and biomedical applications (i.e., wound dressings) require antibacterial agents with safe and biocompatible properties. The antimicrobial activity of synthesized MgO nanoflakes calcined at 400, 500, and 600 °C was studied at a 5 mg/mL MgO concentration for *E. coli* and *S. aureus*, which are representative of Gram-negative and Gram-positive bacteria. As shown in Table 3, at the lower calcination temperatures, MgO-400 °C and MgO-500 °C exhibited excellent antimicrobial activity against *E. coli* (100% R) and *S. aureus* (100% R). However, at high calcination temperatures, MgO-600 °C inhibited *E. coli* (100% R) more than *S. aureus* (>99.99% R). This phenomenon is related to the size of the MgO particles in the antimicrobial mechanism on bacterial cells. The antimicrobial activity of MgO is governed by several key mechanisms, which are similar to those of CaO [30,31]. Upon hydration and dissociation, MgO increases the pH of the surrounding medium, creating an inhospitable environment for bacteria. The dissociation of MgO releases Mg^2+^ ions, which interact with bacterial cell surfaces, leading to membrane destabilization and the disruption of critical cellular functions, including membrane transport, energy metabolism, and cell division. Moreover, MgO generates reactive oxygen species (ROS) such as hydroxyl radicals (HO•), superoxide anions (O_2_•−), and hydrogen peroxide (H_2_O_2_), which contribute to further damage to bacterial cell membranes, as illustrated in Figure 7. Notably, the antimicrobial test demonstrates that Gram-negative bacteria exhibit greater susceptibility to MgO compared to Gram-positive bacteria. Upon dissociation in aqueous media, MgO generates alkaline conditions and releases Mg^2+^, creating a highly detrimental environment for bacterial cells. The Mg^2+^ ions can interact electrostatically with the negatively charged lipopolysaccharides (LPSs) present in the outer membrane of Gram-negative bacteria, resulting in membrane disruption, leakage of intracellular components, and enhanced permeability, allowing Mg^2+^ to penetrate the cell membrane. Additionally, the ROS produced during this process can further damage the bacterial cell membrane, impairing cellular integrity, inhibiting bacterial growth, and ultimately inducing cell death. Previously, Cai et al. [32] demonstrated that MgO nanoparticles significantly generated ROS in *Ralstonia solanacearum*, as measured by the dichlorofluorescein diacetate (DCFH-DA) assay, causing membrane and organelle damage. Additionally, the accumulation of ROS contributes to antibacterial action by inducing oxidative stress and DNA damage. Similarly, Tan et al. [33] reported that MgO nanoparticles deposited on biomedical titanium induced ROS generation in *E. coli* and *S. aureus*, in which ROS levels increased with the increase in thickness of deposited MgO film, as investigated by DCFH-DA assay. Elevated ROS levels induced heightened oxidative stress in the bacteria, thereby contributing to the strong antibacterial efficacy observed against both Gram-negative and Gram-positive strains. The study attributes this antibacterial performance primarily to the alkaline microenvironment created by the MgO film. Regarding the MgO particle size factor, the smaller particles (higher surface area) can more readily dissociate in aqueous media, interact with bacterial cell surfaces, and penetrate the cells, causing membrane destabilization and the disruption of essential cellular functions, leading to cell death. As a result, the smaller MgO particles synthesized from the lower calcination temperatures (400 and 500 °C) exhibited a better antimicrobial efficiency than that obtained from the higher temperature (600 °C). Likewise, Ajitha et al. [34] studied the antibacterial properties of silver nanoparticles with different particle sizes. Their results indicated that the bactericidal effectiveness of silver nanoparticles increased as the particle size decreased.

### 2.6. Viability of RAW 264.7 Macrophage Cells

**Figure 8:**
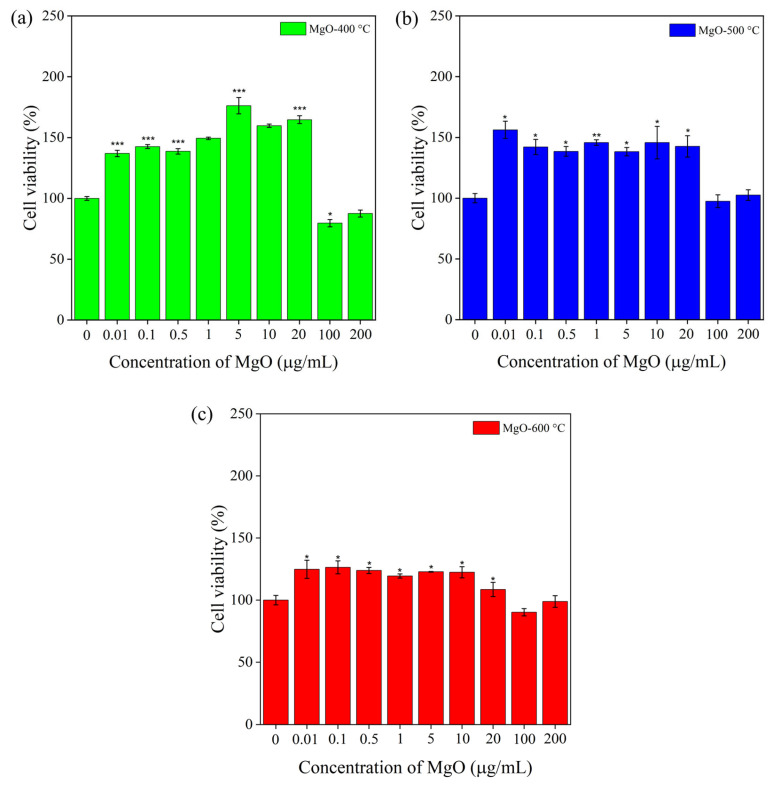
Cell viability (%) of RAW 264.7 macrophages exposed to MgO nanoflakes at varying concentrations and calcination temperatures ((**a**) 400 °C, (**b**) 500 °C, and (**c**) 600 °C). Compared to the control: * *p* < 0.05, ** *p* < 0.01, *** *p* < 0.001, as indicated.

Cytotoxicity testing is essential for determining the potential of MgO in food packaging and biomedical applications. The effect of calcination temperatures (400 °C, 500 °C, and 600 °C) on cell viability (%) of RAW 264.7 macrophages exposed to MgO at varying concentrations (0.01, 0.1, 1, 5, 10, 20, 100, and 200 µg/mL) is illustrated in Figure 8. As shown in Figure 8a, the cell viability of MgO-400 °C significantly increased at lower concentrations of 0.01–20 µg/mL by 137–176%, with the remarkable 176% increase in cell viability at 5 µg/mL indicating a proliferative effect. In contrast, at higher concentrations of 100 and 200 µg/mL, the cell viability of MgO-400 °C was 80 and 88%, respectively. As shown in Figure 8b, MgO-500 °C demonstrated an increase in cell viability, reaching 156% at a low concentration of 0.01 µg/mL. In the concentration range of 0.1–20 µg/mL, cell viability remained between 138% and 146%. At higher concentrations of 100 and 200 µg/mL, MgO-500 °C exhibited 98% and 103% cell viability, respectively, indicating non-cytotoxicity and biocompatibility. As illustrated in Figure 8c, the MgO-600 °C sample is distinct from the MgO-400 °C and MgO-500 °C samples. The cell viability of MgO-600 °C remained relatively stable, ranging from 109% to 126% across concentrations of 0.01 to 20 µg/mL, indicating a slight increase in cell viability at lower doses. At higher concentrations (100–200 µg/mL), MgO-600 °C showed 99-90% cell viability, which slightly reduced by 1–10% compared to the control. For the analysis of in vitro cytotoxicity, cell viability is generally evaluated based on the following criteria compared to the control: non-cytotoxicity (>90%), slight cytotoxicity (60–90%), moderate cytotoxicity (30–59%), and severe cytotoxicity (<30%) [35]. This test confirms that all the synthesized MgO nanoflakes are biocompatible at low concentrations (0.1–20 µg/mL). However, this indicates slight cytotoxicity in MgO-400 at high concentrations (100–200 µg/mL). Notably, the test verified that MgO-500 °C and MgO-600 °C samples are biocompatible and non-cytotoxic, respectively. Based on this observed study, the cytotoxicity of synthesized MgO nanoflakes strongly depends on the particle size, surface area, and concentration. At the same concentration, the MgO with a smaller particle size easily dissociates in aqueous media and releases a greater Mg^2^⁺ ion, which readily reacts with the RAW cell because of its large quantity and surface area compared to the MgO with a larger particle size. In this study, at low concentrations (0.01–20 µg/mL), MgO-400 °C enhanced cell growth and proliferation because of its smaller particle size and higher surface area, which improve cellular uptake and aqueous dissociation, thereby promoting greater Mg^2^⁺ ion release and acting as a mineralization agent for cell growth [36]. The Mg^2^⁺ release capability of MgO plays a vital role in cell growth and metabolic processes, including enzyme activation (as a cofactor for over 300 enzymes), ATP production, protein synthesis, and the maintenance of nucleic acid stability [37,38]. However, MgO-500 °C and MgO-600 °C showed lower cell growth due to their larger particle size and lower surface area, which lower aqueous dissociation, Mg^2^⁺ ion release, and cellular uptake, leading to less cell proliferation [39]. This implies that all synthesized MgO nanoflakes generate a low ROS level at lower concentrations (0.01–20 µg/mL), which are insufficient to generate toxicity to RAW cells but support cell growth. On the contrary, at high concentrations (100–200 µg/mL), MgO-400 °C with smaller particles exhibits slight cytotoxicity to RAW cells due to easy aqueous dissociation, greater Mg^2^⁺ release, and the generation of high ROS levels, leading to oxidative stress, mitochondrial damage, and reduced cell viability. In contrast, MgO-500 °C and MgO-600 °C have diminished dissociation because of their larger particle size and lower surface area, leading to lower ROS production. Consequently, MgO-500 °C and MgO-600 °C demonstrate biocompatibility and non-cytotoxic effects, respectively.

Ge et al. [40] reported that at a 200 mg/mL concentration, spherical MgO nanoparticles with 100 nm particle size were non-toxic to human umbilical vein endothelial cells, while at >500 mg/mL concentrations, the MgO nanoparticles showed toxicity. Mazaheri et al. [41] investigated the toxicity of MgO (10–15 nm) in Wistar rats. They found that white blood cells, red blood cells, hemoglobin, and hematocrit were significantly increased by 250 and 500 µg/mL concentrations in comparison to the control group. As a result, concentrations lower than 250 µg/mL of MgO were suggested as non-toxic and safe for use. Mittag et al. [42] investigated the effect of MgO nanoparticles, ranging in size from 50 to 70 nm, at concentrations from 0.001 to 100 μg/mL on the HT29 intestinal cell line. The MgO showed no cytotoxic or genotoxic effects in HT29 cells and did not induce apoptotic processes, cell cycle changes, or oxidative stress. Patel et al. [43] investigated the effect of spherical and hexagonal MgO (30 nm) on the human intestinal cell line (INT407) and human cervical cancer cell line (SiHa). Their results showed no significant cytotoxicity effect of MgO at concentrations up to 350 µg/mL on the INT407 cell line and up to 250 µg/mL on the SiHa cell line. The current findings on MgO-500 °C and MgO-600 °C demonstrate biocompatibility and non-cytotoxicity, respectively, and are correlated with previous studies. Accordingly, the optimized synthesis parameters for MgO nanoflakes can be leveraged to facilitate their potential applications in wound dressing, drug delivery, nutritional supplement formulations, and active food packaging technologies.

## 3. Materials and Methods

### 3.1. Materials

Magnesium nitrate hexahydrate (Mg(NO_3_)_2_·6H_2_O) and sodium hydroxide (NaOH) were sourced from KemAus, Perth, Australia and RCI Labscan, Bangkok, Thailand. The deionized water and ethanol were obtained from Northern Chemical, Chiang Mai, Thailand. All chemicals were of analytical reagent grade.

### 3.2. Synthesis of MgO

Magnesium nitrate hexahydrate (Mg(NO_3_)_2_·6H_2_O) was separately dissolved in deionized water to prepare a solution with a concentration of 0.2 M. The 2 M sodium hydroxide (NaOH) solution was then gradually introduced dropwise to the reaction mixture, followed by stirring for 90 min at a temperature of 80 °C. The mixture was aged for 24 h, washed with deionized water and ethanol, and dried in an oven at 80 °C for 4 h. The obtained powder was calcined at 400 °C, 500 °C, and 600 °C for 3 h to form MgO nanoflakes, labeled as MgO-400 °C, MgO-500 °C, and MgO-600 °C, respectively.

### 3.3. Characterizations of MgO

#### 3.3.1. XRD Analysis

XRD patterns of MgO nanoflakes were obtained using a Rigaku Miniflex (Rigaku Corporation, Tokyo, Japan) with CuKα (30 kV, 15 mA, λ = 1.542 Å) radiation.

#### 3.3.2. Morphology and Elemental Composition

The morphology, particle size, and elemental composition of MgO nanoflakes were analyzed using a JSM-IT800 FE-SEM with EDS (JEOL Ltd., Tokyo, Japan). The sample was sputtered with gold for 20 s before analysis. A Tecnai G2 20 S-TWIN high-resolution transmission electron microscopy (HR-TEM) (FEI Company, Hillsboro, OR, USA) at 200 kV was used to confirm sample morphology and particle size.

#### 3.3.3. Specific Surface Area

The specific surface area of MgO nanoflakes was measured using a Quantachrome NOVA 2200e BET analyzer, (Boynton Beach, FL, USA).

#### 3.3.4. Thermal Analysis

Thermal stability was analyzed using a Mettler Toledo STARe TGA/DSC3+ thermogravimetric analyzer (Mettler Toledo, Greifensee, Switzerland) in a nitrogen atmosphere (20 mL/min). Around 10 mg of MgO nanoflakes were tested within a temperature range of 0–900 °C at a heating rate of 10 °C/min.

#### 3.3.5. Determination of Antimicrobial Activity

The antimicrobial activity of MgO was assessed using the broth dilution method against Gram-negative (*E. coli*) and Gram-positive bacteria (*S. aureus*) [44]. Bacteria were initially cultured for 18 h, and then 1 mL of bacterial suspension was added to a test tube. Next, 1 mL of MgO solution (5 mg/mL) was introduced into the test tube to achieve a final volume of 2 mL. For the control sample, 1 mL of sterile deionized water was added instead of the MgO solution. The samples were then incubated in a humid chamber at 90% relative humidity and 37 ± 1 °C for 24 h. After incubation, the samples were mixed with peptone solution using a vortex, followed by serial dilution, and loaded onto agar plates. The plates were incubated at 90% relative humidity and 37 ± 1 °C for 24 h. The value of antimicrobial activity (R) was calculated using the following equation:

(1) $$R\;\left(\%\right)=\frac{B-C}{C}\times 100$$

where *B* and *C* are the numbers of colony-forming units (CFUs) of viable microbial cells for the control and MgO samples after 24 h, respectively. All tests were performed in three independent trials.

#### 3.3.6. Cytotoxicity

The RAW 264.7 macrophage cells were cultured in Dulbecco’s Modified Eagle’s Medium (DMEM) with 10% fetal bovine serum (FBS) at 37 °C in a 5% CO_2_ humidified atmosphere. Cells were seeded at a density of 5 × 10^4^ cells/mL in a 96-well plate and incubated for 24 h. The culture supernatant was replaced with fresh medium containing 100 μL of control or MgO at concentrations of 0.01 0.1, 0.5, 1, 5, 10, 20,100, and 200 µg/mL, followed by another 24 h incubation. Cell viability was assessed using the EZCytox assay kit (DoGenBio, Seoul, Republic of Korea) and quantified at 450 and 600 nm with a microplate spectrophotometer, Epoch 2 (BioTek Instruments, Winooski, VT, USA) [36,45].

### 3.4. Statistical Analysis

A statistical analysis was performed by replicating the experiments and calculating the mean and standard deviation (SD) of the data. To compare the means between the two datasets, a paired *t*-test was utilized, with statistical significance established at *p* ≤ 0.05. The analysis was conducted using SPSS Statistics software, version 8 (GraphPad Software Inc., San Diego, CA, USA).

## 4. Conclusions

In the present study, MgO nanoflakes were synthesized via the co-precipitation method with varying calcination temperatures of 400, 500, and 600 °C. The calcination temperatures for the synthesis of MgO nanoflakes influence their particle size, crystallite size, and surface area, which affect the properties of the MgO nanoflakes. All synthesized MgO nanoflakes exhibited a Face-Centered Cubic (FCC) crystalline structure. The crystallite and particle sizes enlarged, while the specific surface area diminished with the rising calcination temperature because crystallites and nanograins tend to aggregate into larger particles during growth, affected by high surface energy and inter-particle electrostatic forces at elevated calcination temperatures. Moreover, MgO synthesized at higher calcination temperatures demonstrated enhanced thermal stability and crystallinity. MgO nanoflakes synthesized at lower calcination temperatures (400 and 500 °C) exhibited superior antimicrobial activity against *E. coli* and *S. aureus* compared to that obtained at the higher calcination temperature (600 °C). The cytotoxicity of MgO nanoflakes strongly depends on their concentration, particle size, and surface area. Notably, cytotoxicity assays revealed that MgO-500 °C and MgO-600 °C exhibited biocompatibility and non-cytotoxic effects, respectively. These findings highlight their promising potential for practical applications such as wound dressing, drug delivery, and active food packaging.

## Figures and Tables

**Table 2:** The element composition of synthesized MgO at different calcination temperatures of 400, 500, and 600 °C was analyzed using SEM-EDS.

Sample	Element	Weight (%)	Atomic (%)
MgO-400 °C	Mg	49.37	39.09
	O	50.63	60.91
MgO-500 °C	Mg	51.47	41.11
	O	48.53	58.89
MgO-600 °C	Mg	60.16	49.84
	O	39.84	50.16

**Table 3:** Antimicrobial activity of synthesized MgO at different calcination temperatures of 400, 500, and 600 °C against *Escherichia coli* and *Staphylococcus aureus*.

Samples	*Escherichia coli*		*Staphylococcus aureus*	
	Variable Cell (CFUs/mL)	R (%)	Variable Cell (CFUs/mL)	R (%)
Control	5.0 × 10^8^	-	7.0 × 10^8^	-
MgO-400 °C	ND	100	ND	100
MgO-500 °C	ND	100	ND	100
MgO-600 °C	ND	100	4.0 × 10^3^	>99.99

ND = Variable bacteria were not detected in plate 10^0^ (volume of 10 μL) at a MgO concentration of 5 mg/mL.

## Data Availability

The original contributions presented in this study are included in the article. Further inquiries can be directed to the corresponding author.
